# On the Treatment of Hæmoptysis

**Published:** 1856-03

**Authors:** 


*On the treatment of Hæmoptysis.* By M. ARAN.

M. Aran agrees with those who entirely condemn the employment of blood-letting in the treatment of hæmoptysis, as it only temporarily arrests the bleeding, while it is dangerous, owing to the debility, and increased susceptibility, to the intercurrent affections it gives rise to. He has, for some time past, been engaged in testing the efficacy of the various hæmostatic agents employed in hæmoptysis; and in this paper he gives the results of his observations. He considers the essence of turpentine a most valuable remedy, given in doses of from 10 to 30 drops every hour, either in a spoonful of water, or mixed up with magnesia as a bolus. Marked amendment usually occurs in a few hours, and in from twenty-four to thirty-six hours the bleeding ceases. It is less suitable for young or plethoric subjects with febrile action, than in weak cachectic individuals, exhibiting atonic characteristics. Ergot of rye and ergotine are far less efficacious; but chloride of sodium, given in doses of 1 to 2½ drachms, proves very efficacious in some cases, and has the advantage of being always at hand. Among the astringents, tannin, and especially gallic acid, are to be recommended; the latter, while quite as efficacious, does not exert the same desiccating effect upon the tissues, or induce the obstinate constipation produced by tannin. As a mean dose, M. Aran gives 15 centigrammes (a centigramme is $\frac{1}{7}$ grain) every hour or alternative hour. He has had little experience in the use of emetic and nauseating remedies; but in three cases in which veratrine was employed, the bleeding ceased as if by enchantment. This class of remedies, indeed, would deserve to stand in the first class of hæmostatic agents, were there not others possessing like efficacy, and yet not giving rise to the painful nausea these produce. M. Aran has derived great advantage from the combined use of digitalis and nitre. In ordinary cases, he gives in the twenty-four hours, 30 centigrammes of digitalis, and 1½ gramme (a gramme is 15 grains) of nitre, divided into four doses; but in very severe cases, these doses may be very much increased, so that the digitalis has been given to the extent of 1½ gramme, and the nitre to 4 grammes, without injuriously affecting the action of the heart, while the effect produced on the hemorrhage has been remarkable. Its arrest, never, however, takes place so suddenly under the use of these medicines, as when turpentine or gallic acid is employed.

In abundant, but not immediately dangerous hemorrhage we can choose among any of the above-mentioned means. In extremely abundant hemorrhage, we must arrest the flow as speedily as possible, by agents which do not depress the powers of the economy too much, and which are not too slow in their operation. Neither ergot, acetate of lead, nor alum is sufficient to meet the danger. Turpentine, gallic acid, chloride of sodium, or nitre with digitalis, can alone be trusted; but the necessity of increasing the dose with the intensity of the hemorrhage may, perhaps, render the chloride of sodium, and especially the nitre and digitalis, dangerous, through the possibility of the production of a too great depression of the heart's action. It is, therefore, to gallic acid or turpentine that we must chiefly trust in these severe cases; and we must not limit ourselves to their employment, but also endeavor to procure a temporary arrest of the hemorrhage by ligatures to the limbs and the application of ice to the chest, allowing the means employed internally to consolidate this temporary cure.—*Med. Times & Gaz., Jan.* 1856, *from Gaz. Hôp.* 1855.

